# Comparing novel treatments in chronic spontaneous urticaria: A critical appraisal of Bruton's tyrosine kinase inhibitors versus anti‐cytokine biologics in clinical trials

**DOI:** 10.1002/clt2.70053

**Published:** 2025-03-27

**Authors:** Anastasia Diamanti, Chiara Tontini, Silvia Bulfone‐Paus

**Affiliations:** ^1^ Lydia Becker Institute of Immunology and Inflammation Division of Musculoskeletal and Dermatological Sciences School of Biological Sciences Faculty of Biology, Medicine and Health University of Manchester Manchester UK

**Keywords:** anti‐cytokine antibodies, biologics, BTK inhibitors, Chronic spontaneous urticaria, randomized clinical trials

To the Editor,

We have been following recent clinical trials focusing on new therapies to treat Chronic Spontaneous Urticaria (CSU). While current treatments can manage the debilitating symptoms, a significant number of patients do not achieve symptom control with available medications, including anti‐IgE treatment.^1^, ^2^ New potential solutions to address this include Bruton's tyrosine kinase (BTK) inhibitors, targeting downstream signaling of the Immunoglobulin E high‐affinity receptor,^3^, ^4^ and anti‐cytokine biologics, targeting T2/alarmin‐driven responses.^5^ However, available literature provides limited comparisons between newer CSU treatments.

We critically appraised the efficacy and safety of six randomized controlled clinical trials (RCTs), three focusing on BTK inhibitors and three on cytokine blockers, to provide insights into the more promising therapeutic options for CSU. Using the Arksey and O'Malley's framework, we systematically reviewed relevant trials. Our search across Embase and Ovid identified 473 studies from April 2019 to May 2024. A secondary search on ClinicalTrials.gov yielded 123 trials, 15 focusing on BTK inhibitors and cytokine blockers. Based on the CONSORT checklist for methodological quality,^6^ we selected trials scoring a minimum of 10 positive or partially met items out of 25. Six RCTs and two pharmaceutical releases (for trial NCT05107115, whose results were not published at the time of the search) were chosen (Figure 1A, Table 1).

**FIGURE 1:**
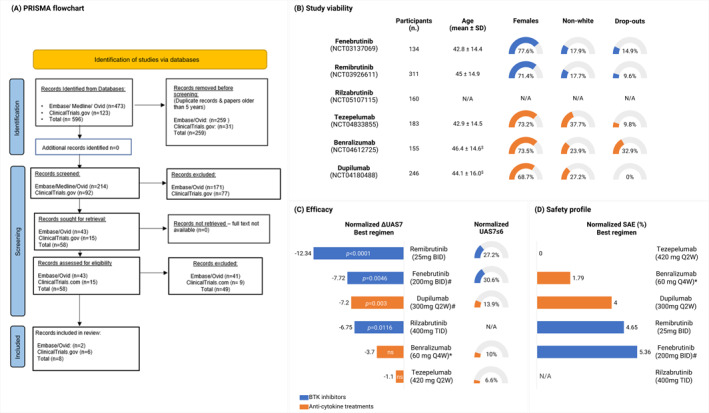
The promising outlook of Bruton's Tyrosine Kinase (BTK) inhibitors and anti‐cytokine strategies to treat chronic spontaneous urticaria. (A) PRISMA diagram of identified and screened records. Eligibility was assessed based on the CONSORT 2010 checklist. (B) Demographic and non‐demographic metrics across placebo and treated cohorts were used to assess the external validity of the different studies. Where not provided by the study authors, the weighted average and standard deviation were calculated ($). (C) Efficacy of the best treatment arm measured using the Urticaria Activity Score over 7 days (UAS7). Results are presented as the net difference in UAS7 scores between the end of the study (EOS) and baseline, normalized by subtracting the scores obtained by the placebo group (ΔUAS7). Percent of subjects achieving complete symptom control (UAS7 ≤6 at EOS) was calculated as the percentage of treated subjects in the best treatment arm and normalized by subtracting the percent observed in the placebo arm. (D) Percent of reported serious adverse events (SAE) in the best treatment arm normalized by placebo. #Average of two cohorts using the same dose regimen. *Participants received Benralizumab 60 mg Q4W until week 12, then 30 mg Q4W until week 24, followed by 30 mg Q4W or Q8W until week 52. BID, twice a day; N/A, information not available; Q2W, every 2 weeks; SAE, serious adverse events; TID, three times a day.

**TABLE 1:** Summary of the trials included in the critical appraisal.

Sponsor	Clinical trial phase	Drug mode of action	Investigated drug	Cohort	Dosage protocol	Participants (*N*)	% Dropouts	% Female participants	% Non‐white participants	Mean age (SD)	Normalized ΔUAS7	*p*‐value ΔUAS7	% Serious adverse effects	Published results (DOI)
Trial identifier: NCT03137069														
Genentech, Inc	Phase 2	‐	Placebo	‐	‐	13	7.70%	84.60%	23.10%	43.6 (11.0)	‐	‐	‐	10.1038/s41591‐021‐01537‐w
		BTK inhibitor	Fenebrutinib	Cohort 1	200 mg BID	28	21.40%	78.60%	14.30%	41.3 (15.9)	−4.89	0.0559	10.71%	
		‐	Placebo	‐	‐	23	13.00%	73.90%	21.70%	40.2 (14.7)	‐	‐	‐	
		BTK inhibitor	Fenebrutinib	Cohort 2	50 mg QD	23	26.10%	78.30%	17.40%	45 (13.10)	−4.44	0.8892	0	
		BTK inhibitor	Fenebrutinib	Cohort 2	150 mg QD	24	8.30%	83.30%	4.20%	43.3 (16.7)	−5.8	0.0717	0	
		BTK inhibitor	Fenebrutinib	Cohort 2	200 mg BID	23	8.70%	69.60%	30.40%	44.3 (13.0)	−10.55	**0.0097**	0	
Trial identifier: NCT03926611														
Novartis pharmaceuticals	Phase 2b	‐	Placebo	‐	‐	43	11.60%	58.10%	18.60%	45.1 (15.2)	‐	‐	‐	10.1016/j.jaci.2022.08.027
		BTK inhibitor	Remibrutinib	‐	10 mg QD	44	6.80%	79.50%	18.20%	42.5 (16.0)	−10.24	**<0.0001**	2.27%	
		BTK inhibitor	Remibrutinib	‐	35 mg QD	44	6.80%	68.20%	15.90%	44 (16.5)	−10.1	**0.0001**	0	
		BTK inhibitor	Remibrutinib	‐	100 mg QD	47	4.30%	83.00%	14.90%	45.2 (13.4)	−7.4	**0.0027**	0	
		BTK inhibitor	Remibrutinib	‐	10 mg BID	44	9.10%	72.70%	18.20%	46.1 (15.2)	−9.8	**0.0002**	4.55%	
		BTK inhibitor	Remibrutinib	‐	25 mg BID	44	9.10%	72.20%	18.20%	47.4 (14.6)	−12.34	**<0.0001**	4.65%	
		BTK inhibitor	Remibrutinib	‐	100 mg BID	45	20.00%	64.40%	20.00%	44.9 (13.8)	−9.51	**0.0003**	0	
Trial identifier: NCT03926611														
Sanofi	Phase 2	‐	Placebo	‐	‐	40	N/A	N/A	N/A	N/A	N/A	N/A	N/A	10.1016/j.jaci.2023.11.893 (Abstract only)
		BTK inhibitor	Rilzabrutinib	‐	400 mg QD	40	N/A	N/A	N/A	N/A	N/A	N/A	N/A	
		BTK inhibitor	Rilzabrutinib	‐	400 mg BD	40	N/A	N/A	N/A	N/A	N/A	N/A	N/A	
		BTK inhibitor	Rilzabrutinib	‐	400 mg TID	40	N/A	N/A	N/A	N/A	−6.75	**0.0116**	N/A	
Trial identifier: NCT04833855														
Amgen	Phase 2	‐	Placebo	‐	‐	48	10.40%	77.10%	39.60%	42.8 (15.2)	‐	‐	‐	10.1016/j.jaci.2023.11.894 (Abstract only)
		Anti‐IgE	Omalizumab	‐	300 mg SC Q4W	31	6.50%	74.20%	45.20%	39.8 (13.1)	‐	‐	‐	
		Anti‐TSLP	Tezepelumab	‐	210 mg SC Q4W	52	13.50%	67.30%	34.60%	45 (14.7)	0.1	0.99	1.92%	
		Anti‐TSLP	Tezepelumab	‐	420 mg SC Q2W	52	7.70%	75.00%	34.60%	42.9 (14.5)	−1.1	0.6	0	
Trial identifier: NCT04612725														
AstraZeneca	Phase 2b	‐	Placebo	‐	‐	40	40.00%	62.50%	25.00%	47.1 (14.3)	‐	‐	‐	10.1093/bjd/ljae067
		Anti‐IL‐5R	Benralizumab	‐	30 mg SC Q4W	59	37.30%	74.60%	22.00%	46.8 (14.4)	−2.6	0.3314	5.08%	
		Anti‐IL‐5R	Benralizumab	‐	60 mg SC Q4W	56	23.20%	80.40%	25.00%	45.6 (15.1)	−3.7	0.1582	1.79%	
Trial identifier: NCT04180488														
Sanofi	Phase 3	‐	Placebo	‐	‐	68	0%	73.50%	29.40%	41.9 (14.8)	‐	‐	‐	10.1016/j.jaci.2024.01.028
		Anti‐IL‐4Rα	Dupilumab	Group A	300 mg SC Q2W	70	0%	58.60%	32.90%	40.7 (16.2)	−8.5	**0.0003**	4%*	
		‐	Placebo	‐	‐	54	0%	75.90%	24.10%	46.8 (16.3)	‐	‐	‐	
		Anti‐IL‐4Rα	Dupilumab	Group B	300 mg SC Q2W	54	0%	68.50%	20.40%	44.1 (15.6)	−5.9	**0.039**	4%*	

*Note*: In bold, statistically significant *p*‐values (*p* < 0.05), *average of all dupilumab‐treated patients (*n* = 124) as provided by the study authors.

Abbreviations: BID, twice a day; BTK, Bruton's Tyrosine Kinase; DOI, digital object identifier; IL‐4Rα, Interleukin 4 receptor alpha chain; IL‐5R, Interleukin 5 receptor; N/A, information not available; Q2W, every 2 weeks; SC, subcutaneous; TID, three times a day; TSLP, thymic stromal lymphopoietin.

Common inclusion criteria included CSU duration of at least 6 months and non‐response to second‐generation H1‐antihistamines, while exclusion criteria included recent infections, other dermatological conditions, immunocompromised status, and major health conditions.

Selected studies were compared across three areas: (A) study characteristics and demographic information (e.g., number of participants, age, gender distribution, ethnic diversity, and dropout rates); (B) CSU outcome measures (e.g., Urticaria Activity Score over 7 days, UAS7); and (C) frequency of serious adverse events.

Data were normalized for the placebo effect, while statistical differences were as reported by the original authors. Studies NCT03137069 and NCT04180488 involved two cohorts with similar dosage protocols, respectively fenebrutinib 200 mg BID and dupilumab 300 mg SC Q2W. Combined *p* values were calculated using Fisher's combined probability test, and the survival function of the chi‐squared distribution was computed using the chi2.sf function of the scipy Python library.^7^ In studies with multiple dosages, we selected the most effective regimen, resulting in the most significant decrease of UAS7 scores from baseline to endpoint normalized to placebo (Normalized ΔUAS7 = [(UAS7_End of study_ – UAS7_Baseline_)]_Treatment_ – [(UAS7_End of study_ – UAS7_Baseline_)]_Placebo_).

Given the randomized controlled design and registration in global clinical trial registries, we considered the internal validity established. In contrast, the external validity was assessed based on demographic/non‐demographic variables representing populations across studies. Out of 6 selected studies, 5 provided sufficient information on clinical trial methods and results for a validity assessment (Figure 1B, Table 1). Although preliminary reports on trial results were accessible,^8^, ^9^ the complete methodology for rilzabrutinib/NCT05107115 was unpublished at the time, thus limiting our analysis. Across the studies, the average dropout rate was highest in benralizumab/NCT04612725 (32.9%) and lowest in dupilumab/NCT04180488 (0%). Participant numbers ranged from 134 to 311, with a mean age of 42.8–46.4 years and a female majority (68.7%–77.6%). Racial composition varied significantly, with non‐white participants ranging from 17.7% to 37.7%. The studies maintained uniform characteristics across placebo and treatment groups, ensuring acceptable validity. However, potential biases could arise from slight differences in dropout rates, gender and racial composition across cohorts, especially in the fenebrutinib/NCT03137069 and dupilumab/NCT04180488 trials.

For instance, the placebo group included a higher percentage of female participants (84.6% Cohort 1, 73.9% Cohort 2) compared to the 200 mg BID‐treated arms of the fenebrutinib trial (78.6% Cohort 1, 69.6% Cohort 2) and in placebo (73.5% Group A, 75.9% Group B) versus dupilumab 300 mg Q2W (58.6% Group A, 68.5% Group B). Similarly, drop‐out rates differed notably between the fenebrutinib 200 mg BID‐treated cohorts (21.4% Cohort 1 vs. 8.7% Cohort 2). These discrepancies may have also contributed to the more significant UAS7 reduction observed in the respective treated arms.

When evaluating efficacy, four medications produced statistically significant improvement in CSU symptoms. Remibrutinib administered 25 mg BID proved most effective, with ΔUAS7 equal to −12.34 (*p* < 0.0001), while fenebrutinib (200 mg BID), rilzabrutinib (400 mg TID) and dupilumab (300 mg Q2W) ranged from −7.72 to −6.75 (Figure 1C). Fenebrutinib also displayed the highest rate of well‐controlled CSU (30.6% UAS7 ≤6). On average, BTK inhibitor‐based treatments offered a 19% greater improvement in ΔUAS7 compared to dupilumab and 55% compared to all cytokine blockers combined. However, anti‐cytokine treatments reported less severe adverse events (0%–4%) than BTK inhibitors (4.65%–5.36%, Figure 1D). Overall, remibrutinib and dupilumab emerged as the best options per each drug category, with the highest efficacy and relatively lower frequency of adverse events.

In conclusion, our analysis of phase 2 RCTs on BTK inhibitors for CSU treatment demonstrated higher efficacy in symptom control, although results from ongoing phase 3 RCTs are still warranted. Except for dupilumab, none of the analyzed studies addressed treatment response by CSU subtype (i.e., type I vs. type IIb autoimmune CSU) or stratified by anti‐IgE treatment response. Hence, it remains unclear whether underperforming treatments, like anti‐TSLP and IL‐5 receptor antibodies, may offer superior outcomes in specific patient groups and/or associated comorbidities.^10^

## AUTHOR CONTRIBUTIONS

Anastasia Diamanti and Silvia Bulfone‐Paus conceptualized the study. Anastasia Diamanti performed the database search and statistical analyses. Anastasia Diamanti and Chiara Tontini created the figure and table. Anastasia Diamanti, Chiara Tontini and Silvia Bulfone‐Paus wrote and revised the manuscript.

## CONFLICT OF INTEREST STATEMENT

The authors declare no conflicts of interest.

## Data Availability

Data available on request from the authors.
